# Experiences and Perceptions of Interprofessional Collaboration Among Spanish Optometrists and Occupational Therapists: A Cross‐Sectional Study

**DOI:** 10.1155/oti/7088426

**Published:** 2026-08-10

**Authors:** María José López-de-la-Fuente, Víctor Berdejo, Laura Gonzalo-Ciria, Carmen Bilbao, Carmen López-de-la-Fuente

**Affiliations:** ^1^ Department of Physiatry and Nursing, Faculty of Health Sciences, University of Zaragoza, Zaragoza, Spain, unizar.es; ^2^ iHealthy Research Group, Aragon Health Research Institute (IIS Aragón), Zaragoza, Spain; ^3^ Department of Applied Physics, Faculty of Science, University of Zaragoza, Zaragoza, Spain, unizar.es; ^4^ Department of Physical Therapy, Occupational Therapy, Rehabilitation and Physical Medicine, Faculty of Health Sciences, Rey Juan Carlos University, Alcorcón, Spain, urjc.es; ^5^ Department of Ophthalmology, Hospital Universitario Miguel Servet, Zaragoza, Spain, oftalmar.es

**Keywords:** attitude of health personnel, interprofessional collaboration, interprofessional relations, occupational therapy, optometry

## Abstract

**Introduction:**

Interprofessional collaboration (IPC) between optometrists and occupational therapists is key to optimising care for people with visual and functional difficulties. This study examines the experiences and perceptions of IPC among both disciplines in Spain, identifies factors shaping collaboration and compares actual collaborative practice with perceived IPC priorities.

**Methods:**

A cross‐sectional study was conducted using an online survey. Participants, 138 optometrists and 148 occupational therapists, were categorised into two groups: those with prior collaborative experience (G1 = 92) and those without (G2 = 194). The survey included sociodemographic data, practice fields and perceptions of IPC. Drawing on D′Amour′s model, the dimensions of shared goals and vision, internalisation, formalisation and governance—and their related indicators—were analysed. Quantitative data were examined using descriptive statistics, chi‐square tests, Fisher′s exact tests, Mann–Whitney U tests and Kruskal–Wallis tests. Open‐ended responses were analysed descriptively.

**Results:**

Only 32.2% of participants reported prior IPC experience, and collaboration was more frequent in paediatric and private settings. Many G2 professionals indicated serving clients with low vision or acquired brain injury without collaborating, highlighting missed opportunities. G1 participants reported a positive impact on practice and professional learning and scored highly in internalisation, indicating trust and mutual understanding. Nevertheless, gaps persisted in shared goals, formalisation and governance. Occupational therapists reported a more client‐centred approach than optometrists and perceived some role‐boundary barriers. Limited role awareness and uncertainty about referral pathways were expressed by G2. Both groups noted a need for targeted training to support IPC.

**Conclusions:**

IPC between optometrists and occupational therapists in Spain is currently limited and mainly informal. Prior collaborative experience was associated with greater trust and clearer role understanding. Interprofessional education, clear referral pathways and organisational support are essential to strengthen IPC and ensure client‐centred, comprehensive care for individuals with visual impairment.

## 1. Introduction

Visual impairments affect a wide range of clinical populations. In Spain, glaucoma, diabetic retinopathy, age‐related macular degeneration and high myopia cause low vision, predominantly in older adults, and represent a high cost to the healthcare system [1]. Low vision is associated with significant limitations in daily functioning [2, 3]. Additionally, children with learning disorders [4] and disabilities [5] present visual difficulties that affect academic performance and daily activities. Children with and without neurodevelopmental disorders in Spain experience visual problems, supporting the need for visual assessment in paediatric care [6].

Furthermore, after stroke and traumatic brain injury, visual problems are common, frequently underdiagnosed and significantly impact daily activities and rehabilitation outcomes [7–9]. Despite their prevalence, visual screening is rarely implemented, and vision specialists (e.g., ophthalmologists and optometrists) are frequently absent from neurorehabilitation teams across healthcare systems [10, 11]. Unaddressed visual difficulties may affect an individual′s engagement in meaningful occupations across the lifespan. Therefore, such vision‐related functional challenges require input from occupational therapists and optometrists.

Coordinated care is crucial as health systems confront increasingly complex client needs involving multiple disciplines [12, 13]. The World Health Organization (WHO) identifies interprofessional collaboration (IPC) as a key strategy for strengthening healthcare delivery [14]. IPC does not occur by chance; it requires specific training and shared professional culture to support high‐quality, client‐centred care [12–14]. Practitioners must also recognise when clients need another discipline′s expertise and understand their own roles and scope of practice [7]. In Spain, occupational therapy continues to consolidate its professional identity and faces internal and external role confusion [15], whereas optometry seeks broader recognition of vision therapy within healthcare [16].

Meanwhile, professional bodies in both occupational therapy and optometry highlight the value of IPC for addressing visual and functional needs [17, 18]. Recent clinical guidelines recommend collaboration between occupational therapists and eye care professionals [7, 8]. Evidence from low vision and neurological rehabilitation shows that such collaborative practice is implemented and can support daily functioning and occupational performance [2, 3, 19]. In paediatric contexts, coordinated care is needed to address visual difficulties that affect learning, academic performance and daily activities [6, 20].

Effective IPC, however, requires a clear understanding of each profession′s role. Whilst occupational therapists and optometrists share complementary scopes of practice, their professional perspectives differ. These overlapping areas of intervention, often described as ‘shared spaces’, offer opportunities for interprofessional synergy but may also generate challenges related to professional identity [21]. Occupational therapists are trained to screen some visual issues, with a primary focus on how visual functions, as client factors, interact with occupations and environmental contexts to shape occupational performance [17]. On the other hand, optometrists in Spain focus on the assessment and management of visual function—including refractive errors, accommodative and oculomotor dysfunctions and binocular vision anomalies—to optimise visual efficiency in functional tasks [22]. Operating at Advanced Proficiency Level 3 of the World Council of Optometry, optometrists also deliver vision therapy interventions and prescribe optical or assistive devices for individuals with low vision [22, 23]. Recognising these distinctions, alongside the potential of shared competencies, supports the development of effective collaborative practice.

Despite the potential benefits of IPC, such partnerships remain limited in practice. Evidence suggests that professionals from both disciplines rarely consult one another, reflecting not only fragmented referral pathways and differing clinical approaches but also practical and organisational barriers such as time constraints, insufficient interprofessional training and a lack of shared institutional affiliation [11, 20, 24, 25]. The limited mutual awareness of roles and scopes of practice exacerbates these challenges [11, 26]. Whilst research on IPC among these professions has been conducted internationally, evidence from the Spanish context remains scarce. Greater insight into these context‐specific barriers and facilitators is therefore needed to inform strategies that strengthen IPC. This can help reduce duplicative interventions, promote more efficient service delivery and achieve better client‐centred outcomes.

A theoretical framework is essential for analysing IPC in a structured way. The D′Amour model identifies 10 key indicators grouped into four interconnected dimensions: (1) shared goals and vision, (2) internalisation, (3) formalisation and (4) governance [27]. These indicators encompass personal, relational, organisational and systemic factors that influence collaborative practice. Complementing this model, a recent meta‐review [13] argues that effective IPC is shaped by a dynamic interplay of factors operating at three interdependent levels: individual (e.g., communication skills and role clarity), team‐based (e.g., mutual trust and coordination) and organisational (e.g., leadership support and shared structures). As shown in Table 1, this integrated perspective captures the nuances of collaboration, particularly the dual nature of ‘leadership’, distinguishing between formal authority (organisational) and professional‐led initiative (individual or team).

**Table 1:** Integrated framework for interprofessional collaboration.

Dimension (D′Amour)	Key indicators	Illustrative focus	Predominant level^a^
Shared goals and vision	1. Shared goals	Alignment of values and common goals centred on client′s needs	Individual/team
	2. Client‐centred		

Internalisation	3. Mutual acquaintanceship	Interpersonal relationships, role clarity and professional confidence	Individual/team
	4. Trust		

Governance	5. Centrality	Strategic direction and institutional positioning that legitimise and foster IPC	Organisational/system
	6. Leadership	Formal: Position‐related authority	Individual/team/organisational
		Emergent: Professional‐led initiative	
	7. Support for innovation	Resources and training to implement IPC	Organisational
	8. Connectivity	Structured communication channels and forums for coordination	Organisational

Formalisation	9. Formalisation tools	Referral protocols and agreements	Team/organisational
	10. Information exchange	Active feedback and clinical information to monitor progress	Individual/team

^a^Levels informed by Wei et al. (2022).

IPC has been described as a dynamic continuum rather than a fixed ideal. This continuum ranges from networking (low interdependence) to teamwork (high integration). The level of collaboration is shaped by clinical and organisational settings, care complexity and the ongoing negotiation of professional roles. This perspective underscores the importance of examining how collaboration is enacted in specific contexts [21, 28].

Against this backdrop, this descriptive and exploratory cross‐sectional study examines the experiences and beliefs shaping IPC between optometrists and occupational therapists in Spain. It contrasts actual collaborative experiences with the perceived importance of IPC components among professionals without prior collaborative experience. The study also identifies shared areas of practice, examines the perspectives of both disciplines and highlights aspects of care that could benefit from targeted training or greater coordination. These insights aim to inform professional education, service development and strategies to enhance collaborative care.

The study is aimed at addressing the following research questions:

1. What are the professional and practice characteristics of Spanish occupational therapists and optometrists with and without previous IPC experience, including years of experience, academic qualifications, practice settings and clinical populations served?
2. What relative importance do professionals without prior collaborative experience place on the different components of IPC?
3. How do perceptions of IPC differ across disciplines (optometrists vs. occupational therapists) and between groups (those with prior collaborative experience and those evaluating IPC priorities) regarding collaboration indicators, mutual knowledge and professional boundaries?
4. What challenges and opportunities emerge for strengthening IPC between optometrists and occupational therapists in Spain?

## 2. Materials and Methods

### 2.1. Study Design

This study employed an online cross‐sectional survey design, with a primary quantitative focus and a complementary descriptive analysis of open‐ended responses. The first page of the survey form included information about the study, and respondents provided consent to participate. The form also included links to Google and Zaragoza University′s privacy and data protection policies. The Clinical Research Ethics Committee of Aragon (CEICA) granted ethical approval (CP‐CI PI22/443). The study protocol adhered to the Strengthening the Reporting of Observational Studies in Epidemiology (STROBE) statement [29].

### 2.2. Participants and Data Collection

Snowball and convenience sampling methods were employed to recruit participants. The research team disseminated the survey link through professional associations, forums and social media. Distribution was managed through internal communication channels, and the authors did not have access to individual contact details. Only qualified occupational therapists and optometrists working in Spain, regardless of their field of practice, were eligible to participate. The survey was available from May to July 2024.

### 2.3. Survey Development and Pretesting

Two optometrists and two occupational therapists from the research team designed the initial draft of the six‐section survey. Sections 4 and 5 were based on the collaboration indicators proposed by D′Amour et al. [27]. A previously validated Spanish questionnaire developed by Nuño‐Solinís et al. [30] informed the formulation of the items in these sections. This instrument operationalised D′Amour′s 10 indicators and demonstrated good internal consistency (Cronbach^′^s *α* = 0.866) [30]. More than one item was used to assess most collaboration indicators. The items were worded for the specific professional context of collaboration between optometrists and occupational therapists. No separate psychometric validation was conducted for Sections 4 and 5 before their use in this study.

The survey was reviewed by five occupational therapists and five optometrists, who evaluated its clarity, relevance and completion time. Minor adjustments were made based on their feedback. The questionnaire comprised six sections and was designed for completion in approximately 15 min (the original Spanish version and an English translation are available in the supporting information (Available here)). Based on survey responses, participants were categorised into two groups: those with prior collaborative experience (Group 1 [G1]) and those without (Group 2 [G2]).

Section 1: sociodemographic information. Five single‐choice (profession, gender, age, academic qualification and years of practice) and one multiple‐choice question (work setting) were included.

Section 2: fields of practice. Four multiple‐choice questions explored practice areas, client health issues, age groups served and collaboration with other professions. This section concluded with a dichotomous (yes/no) question used to categorise participants into one of two groups (G1 and G2).

Section 3: collaboration details (G1 only). This section explored the number and years of collaboration, partnership initiation, communication frequency and channels (multiple‐choice), satisfaction with collaboration (5‐point Likert scale), referrals (multiple‐choice, cross‐centre and within‐centre referrals) and case‐based reflection through two open‐ended questions exploring the impact of recent cases on client outcomes and professional growth.

Section 4: IPC indicators (G1 only). Eighteen questions assessed participants′ experiences of collaboration based on the model proposed by D′Amour et al. and Nuño‐Solinís et al. [27, 30]. All items except goal‐setting were rated on a 5‐point Likert scale. One multiple‐choice question examined the distribution of goal‐setting responsibilities (professional‐led, shared with the team or client‐ or family‐led).

Section 5: IPC perceptions (G2 only). This section included corresponding questions to those in Section 4 but asked respondents to rate the relative importance of each item. These questions explored how professionals without prior collaborative experience valued the different components of IPC. This approach aimed to examine the relative importance attributed to IPC indicators in the absence of direct collaboration. The questions assessing role knowledge and role‐boundary protectionism were identical to those used in Section 4.

Section 6: additional feedback (G1 and G2). This section included a dichotomous (yes/no) question on the need for referral protocols, followed by an open‐ended question for further comments.

### 2.4. Data Analysis

Descriptive statistics were calculated for each variable type: qualitative variables as counts and percentages in contingency tables and quantitative or ordinal variables as the median and interquartile range (IQR). Given the ordinal nature of the data and non‐normal distribution, nonparametric tests were applied. Group differences in categorical variables were examined with Pearson′s *χ* ^2^ test; Fisher′s exact test was substituted when expected cell counts were < 5. Independent‐sample quantitative or ordinal scores were compared with the two‐tailed Mann–Whitney *U* test. No missing data were observed; the survey questions included in the quantitative analyses were mandatory. Statistical significance was set at *p* < 0.05, with a statistical power of 80%. Analyses were conducted using R software Version 4.4.2 [31] within the RStudio environment [32].

#### 2.4.1. Visualisation

Graphical representations were used to interpret survey data. A plot was generated to display the frequency and types of communication channels reported by the collaboration group (G1). Normalised bar charts compared collaboration with other professionals across groups and professions. Kiviat (radar) charts were constructed to illustrate participants′ perceptions across the 10 D′Amour indicators, on a scale from minimal (1) to maximal (5). For G1, higher scores indicated greater IPC enactment in daily practice. For G2, higher scores reflected the extent to which an indicator was perceived as important for facilitating IPC in the absence of direct collaboration.

For D′Amour′s indicators, a mean score was calculated by averaging the responses to two related questions for most indicators. Specifically, ‘trust’ was calculated by combining the reversed ‘role protectionism and professional boundaries’ item (higher scores indicating lower trust in G1) with the direct trust question. All scores were treated as ordinal and summarised using the median and IQR. Goal‐setting responses were recoded on a 1–5 scale: 1 = *professional*, 2 = *team* (professional optional), 4 = *client and professional*, 4.5 = *all options* (professional, team, and client), denoting shared but unclear decision‐making, and 5 = *client* (team optional).

Mutual knowledge of roles and role‐boundary perceptions was analysed by group (G1 vs. G2) and profession, using the Mann–Whitney *U* test. Open‐ended responses were analysed descriptively to provide illustrative examples that complemented the findings. Two researchers (M.J.L‐F. and C.L‐F.) reviewed the responses through a consensus‐based approach to identify common remarks and select representative quotes that contextualised the participants′ experiences.

## 3. Results

### 3.1. Participants

The study included 138 optometrists and 148 occupational therapists; most participants were women (87.76%). G1 (collaborating) included 92 professionals, and G2 (noncollaborating) included 194. No significant differences were observed between G1 and G2 regarding gender, years of experience or service provision to adolescent/adult populations. However, when academic qualification was analysed as an ordinal variable, G1 participants showed significantly higher qualification levels than G2 participants (*p* = 0.043; Table 2).

**Table 2:** Descriptive statistics of sociodemographic variables and professional characteristics of participants by group and profession.

	**Total (** **N** = 286 **) (%)**	**G1 (** **n** = 92 **) (%)**	**G2 (** **n** = 194 **) (%)**	**p** **value**	**Optometrist (** **n** = 138 **) (%)**	**Occupational therapists (** **n** = 148 **) (%)**	**p** **value**

Optometrists	138 (48.3)	53 (18.5)	85 (29.7)	0.040^a^			
Occupational therapists	148 (51.7)	39 (13.6)	109 (38.1)				
Gender							
Male	32 (11.2)	10 (3.5)	22 (7.7)	0.74^b^	22 (15.9)	10 (6.8)	0.044^a^
Female	251 (87.8)	82 (28.7)	169 (59.1)		115 (83.3)	136 (91.9)	
Does not declare	3 (1.1)	0 (0)	3 (1.1)		1 (0.7)	2 (1.4)	
Highest academic qualification							
Diploma higher education	81 (28.3)	23 (8.0)	58 (20.3)	0.063^b^	37 (26.8)	44 (29.7)	0.836^a^
Bachelor′s degree	68 (23.5)	15 (5.2)	53 (18.5)		31 (22.5)	37 (25.0)	
Master′s degree	129 (45.3)	51 (17.8)	78 (27.3)		66 (47.8)	63 (42.6)	
Doctoral degree	8 (2.7)	3 (1.1)	5 (1.7)		4 (2.9)	4 (2.7)	
Years of experience							
< 5 years	47 (16.4)	8 (2.8)	39 (13.6)	0.144^a^	15 (10.9)	32 (21.6)	0.006^a^
5–10 years	61 (21.3)	24 (8.4)	37 (12.9)		29 (21.0)	32 (21.6)	
10–15 years	63 (22.0)	20 (7.0)	43 (15.0)		31 (22.5)	32 (21.6)	
15–20 years	38 (13.3)	14 (4.9)	24 (8.4)		14 (10.1)	24 (16.2)	
> 20 years	77 (26.9)	26 (9.1)	51 (17.8)		49 (35.5)	28 (18.9)	
Age groups served							
Paediatrics	213 (74.5)	85 (29.7)	128 (46.8)	< 0.001^a^	135 (97.8)	78 (52.7)	< 0.001^a^
Adolescents/adults	243 (85.0)	76 (26.6)	167 (58.4)	0.555^a^	137 (99.3)	106 (71.6)	< 0.001^a^
Older adults	213 (74.5)	59 (20.6)	154 (53.9)	0.009^a^	122 (88.4)	91 (61.5)	< 0.001^a^

	**Mdn (IQR)**	**Mdn (IQR)**	**Mdn (IQR)**	**p** **value**	**Mdn (IQR)**	**Mdn (IQR)**	**p** **value**

Participants′ age	38.0 (14.0)	38.5 (11)	37.5 (15)	0.256^c^	39.5 (13)	36 (13.5)	0.001^c^
Academic qualification	2 (2.0)	3 (1.25)	2 (2.0)	0.043^c^	3 (2)	2 (2)	0.401^c^

*Note:* Multiple responses were allowed for clients′ age groups served. Statistical significance was set at *p* < 0.05.

Abbreviations: IQR, interquartile range; Mdn, median.

^a^ *p* values were obtained using Pearson′s chi‐squared test.

^b^ *p* values were obtained using Fisher′s exact test.

^c^ *p* values were obtained using the Mann–Whitney *U* test.

Compared with occupational therapists, optometrists were older, had more professional experience, served clients of all age groups more frequently and reported previous collaborative experience more often (*p* = 0.040). Finally, previous IPC experience was more common among professionals serving paediatric clients, whereas those serving older adults were more frequently represented in G2 (Table 2).

### 3.2. Work Settings, Referrals and Ways of Communication

#### 3.2.1. Work Settings

Optometrists predominantly worked in visual care centres or clinics, whereas occupational therapists were distributed across private clinics, hospitals and aged care facilities. Employment in private clinics was more common among G1 participants in both professions, whereas optometrists had a limited presence in hospital settings (Table 3).

**Table 3:** Workplace distribution of study participants.

	Optometrists			Occupational therapists		
	*n* = 138(%)	G1 (*n* = 53) (%)	G2 (*n* = 85) (%)	*n* = 148(%)	G1 (*n* = 39) (%)	G2 (*n* = 109) (%)
Visual care centre/optical shop	101 (73.2)	32 (60.4)	69 (81.2)	2 (1.4)	2 (5.1)	0 (0.0)
Hospital	4 (2.9)	1 (1.9)	3 (3.5)	30 (20.3)	5 (12.8)	25 (22.9)
Private clinics	42 (30.4)	24 (45.3)	18 (21.2)	41 (27.7)	25 (64.1)	16 (14.7)
Aged care facilities	0 (0.0)	0 (0.0)	0 (0.0)	26 (17.6)	2 (5.1)	24 (22.0)
Nonprofit organisations	2 (1.4)	2 (3.8)	0 (0.0)	21 (14.2)	4 (10.3)	17 (15.6)
University	7 (5.1)	2 (3.8)	5 (5.9)	8 (5.4)	3 (7.7)	5 (4.6)
Education	1 (0.7)	1 (1.9)	0 (0.0)	11 (7.4)	3 (7.7)	8 (7.3)
Early intervention centres	0 (0.0)	0 (0.0)	0 (0.0)	13 (8.8)	5 (12.8)	8 (7.3)
Other	0 (0.0)	0 (0.0)	0 (0.0)	23 (15.5)	3 (7.7)	20 (18.3)

*Note:* Multiple responses were allowed; percentages may exceed 100%.

#### 3.2.2. Referrals and Initiation of Collaboration

In G1, 91.3% reported referring clients externally, whereas internal referrals were uncommon and mainly occurred in private clinics (*n* = 8). Most G1 participants collaborated with one to two professionals from the other discipline (66.3%). Collaboration most commonly began through joint work with a client (78.3%), followed by working in the same workspace (17.4%) and attendance at training or conferences (4.3%). Regarding seniority, 71.7% of professionals began collaborating within the past 5 years, although 9.8% reported long‐term relationships of more than 10 years.

#### 3.2.3. Communication Methods

**Figure 1:**
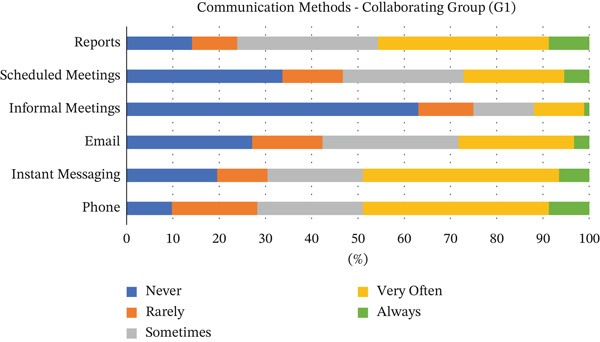
Ways of communication in the collaborating group (G1).

G1 participants primarily communicated via instant messaging, phone calls and reports, whereas email and scheduled meetings were used less often. Informal meetings were the least standard method of interaction (Figure 1).

### 3.3. Field of Practice and Collaboration With Other Professionals

Table 4 shows differences in practice areas and client populations served across disciplines and groups. Among optometrists, G1 participants were more frequently involved in vision therapy, learning disabilities and acquired brain injury (ABI) (*p* < 0.05). G1 occupational therapists worked with children with disabilities, including those with neurodevelopmental or sensory‐processing disorders, more often, with a stronger paediatric focus. Notably, many G2 participants reported serving individuals with low vision or ABI.

**Table 4:** Practice areas and client populations served by profession and group.

		Optometrists (*n* = 138)				Occupational therapists (*n* = 148)			
		Total *n*(%)	G1 *n*(%)	G2 *n*(%)	*p* value	Total *n*(%)	G1 *n*(%)	G2 *n*(%)	*p* value
Low vision	No	102 (73.9)	41 (40.2)	61 (59.8)	0.597	111 (75)	32 (28.8)	79 (71.2)	0.332
	Yes	36 (26.1)	12 (33.3)	24 (66.7)		37 (25)	7 (18.9)	30 (81.1)	
Learning disabilities	No	39 (28.3)	7 (17.9)	32 (82.1)	0.004	87 (58.8)	8 (9.2)	79 (90.8)	< 0.001
	Yes	99 (71.7)	46 (46.5)	53 (53.5)		61 (41.2)	31 (50.8)	30 (49.2)	
Acquired brain injury	No	88 (63.8)	20 (22.7)	68 (77.3)	< 0.001	67 (45.3)	18 (26.9)	49 (73.1)	1
	Yes	50 (36.2)	33 (66)	17 (34)		81 (54.7)	21 (25.9)	60 (74.1)	
Children with disabilities	No	89 (64.5)	28 (31.5)	61 (68.5)	0.038	103 (69.6)	13 (12.6)	90 (87.4)	< 0.001
	Yes	49 (35.5)	25 (51)	24 (49)		45 (30.4)	26 (57.8)	19 (42.2)	
Vision therapy	No	30 (21.7)	6 (20)	24 (80)	0.033				
	Yes	108 (78.3)	47 (43.5)	61 (56.5)					
Binocular vision dysfunction	No	7 (5.1)	0 (0)	7 (100)	0.043^a^				
	Yes	131 (94.9)	53 (40.5)	78 (59.5)					
Strabismus	No	40 (29)	8 (20)	32 (80)	0.008				
	Yes	98 (71)	45 (45.9)	53 (54.1)					
Contact lenses	No	25 (18.1)	13 (52)	12 (48)	0.188				
	Yes	113 (81.9)	40 (35.4)	73 (64.6)					
Neurodevelopmental disorders				No		65 (43.9)	5 (7.7)	60 (92.3)	< 0.001
				Yes		83 (56.1)	34 (41.0)	49 (59.0)	
Neurodegenerative diseases				No		76 (51.4)	24 (31.6)	52 (68.4)	0.195
				Yes		72 (48.6)	15 (20.8)	57 (79.2)	
Mental disorders				No		83 (56.1)	27 (32.5)	56 (67.5)	0.082
				Yes		65 (43.9)	12 (18.5)	53 (81.5)	
Sensory processing disorders				No		79 (53.4)	7 (8.9)	72 (91.1)	< 0.001
				Yes		69 (46.6)	32 (46.4)	37 (53.6)	

*Note:* Multiple responses were allowed.

^a^Fisher′s exact test.

**Figure 2:**
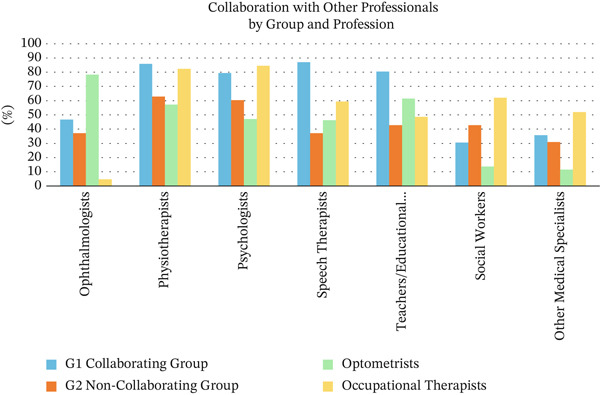
Collaboration with other professionals by group and profession.

Relationships with other professionals also showed differences. Optometrists reported collaborating more frequently with ophthalmologists (78.3%). Occupational therapists collaborated more with psychologists (84.5%), physiotherapists (82.4%) and social workers (62.2%) but rarely with ophthalmologists (4.7%). Reported collaboration with other professionals was generally higher in G1 than in G2 (Figure 2).

### 3.4. D′Amour Indicators

**Figure 3:**
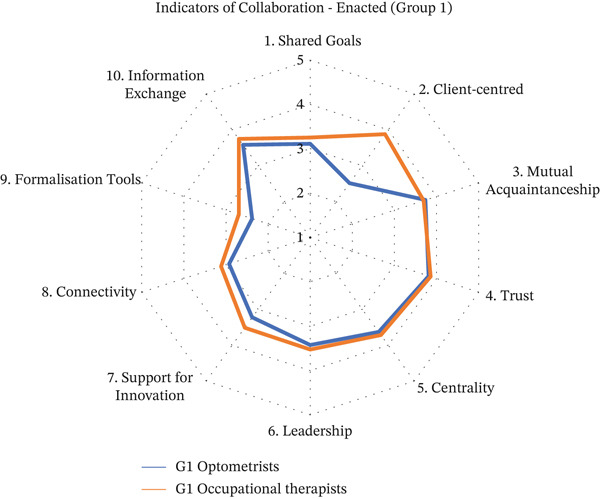
Collaboration indicators—enacted (G1).

**Figure 4:**
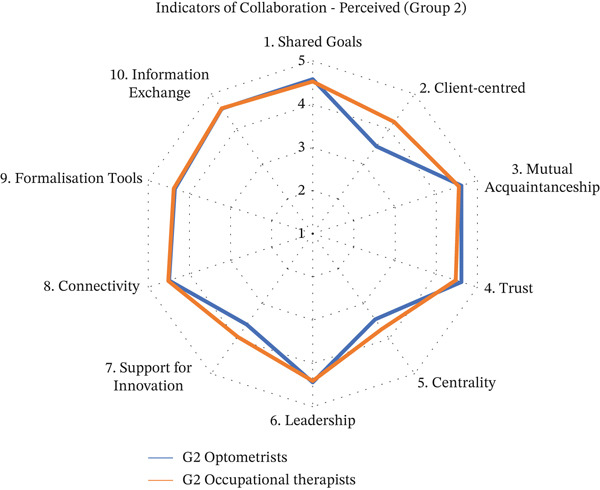
Collaboration indicators—perceived (G2).

Kiviat plots show the D′Amour collaboration profiles for both groups from two perspectives: G1 profiles reflect IPC enactment in daily practice, whereas G2 profiles represent the perceived importance of IPC indicators. Profiles were similar between professions within each group. However, a distinct divergence was observed in the ‘client‐centred’ indicator, where optometrists showed lower scores than occupational therapists in both G1 and G2 (Figures 3 and 4). Overall, G1 and G2 scores differed significantly on most indicators (*p* < 0.001), except ‘centrality’ (*p* = 0.763; Table 5).

**Table 5:** D′Amour′s collaboration indicators (by group) and mutual knowledge and perceived role boundaries (by group and profession).

**Indicator**	**G1 (** **n** = 92 **) Mdn (IQR)**	**G2 (** **n** = 194 **) Mdn (IQR)**	**p** **value**

Shared goals	3 (1.5)	5 (1)	< 0.001
Client‐centred	2 (3)	4 (2)	< 0.001
Mutual acquaintanceship	4 (1)	5 (1)	< 0.001
Trust	4 (1)	5 (1)	< 0.001
Centrality	4 (1.5)	4 (1)	0.763
Leadership	3.5 (1.5)	5 (1)	< 0.001
Support for innovation	3.5 (1.5)	4 (1)	< 0.001
Connectivity	3 (1)	5 (1)	< 0.001
Formalisation tools	2.5 (1.5)	5 (1)	< 0.001
Information exchange	4 (1)	5 (1)	< 0.001

**Role knowledge and professional boundary perceptions**			
	**G1**	**G2**	**p** **value**

Knowledge of roles	4 (1)	3 (2)	< 0.001
Role‐boundary perceptions	2 (1)	4 (1)	< 0.001

	**Optometrists (** **n** = 138 **)**	**Occupational therapists (** **n** = 148 **)**	**p** **value**

Knowledge of roles	4 (1)	3 (1.25)	0.904
Role‐boundary perceptions	2 (2)	4 (1)	0.002

*Note:* Role‐boundary perceptions: extent to which participants perceived the protection of the other profession′s role boundaries as a barrier to collaboration (1 = *not at all*; 5 = *completely*). Higher scores indicate greater perceived barriers.

Abbreviations: IQR, interquartile range; Mdn, median.

### 3.5. Working Together on the Ground: Knowledge, Attitudes and Experiences

G1 reported greater knowledge of the other discipline and saw fewer role‐boundary barriers than G2. No differences between professions were observed for knowledge of roles. However, occupational therapists reported higher role‐boundary perception scores than optometrists (median = 4 vs.2; *p* = 0.002; Table 5).

Open‐ended responses were analysed descriptively to supplement quantitative findings. Four categories described IPC impact, professional learning, operational barriers and unmet needs.

#### 3.5.1. Clinical Impact

G1 participants reported that IPC improved their practice. Collaboration produced holistic, effective interventions and often quickened progress. The survey item on satisfaction with collaboration yielded high scores (median = 4; mode = 5). ‘Collaboration helped me address patients′ needs more effectively’ (optometrist, G1). ‘Working together leads to quicker, more effective interventions’ (occupational therapist, G1). However, concerns about the burden of multiple referrals and financial constraints on families persisted.

#### 3.5.2. Professional Growth and Role Internalisation (G1)

Joint work fostered mutual learning, improved understanding of the other profession′s scope and clarified one′s own limits. An occupational therapist (G1) shared, ‘I have gained a greater understanding of the impact of visual issues on daily functioning’. An optometrist (G1) said, ‘It has enriched my professional knowledge. My vision therapy sessions are more integrative’.

#### 3.5.3. Professional Silos and Informal Collaboration (G1 and G2)

Some G1 participants described failed collaborations, often due to one‐way referrals and poor follow‐up. Many G1 participants felt that collaboration depended on individual initiative rather than on organisational support. G2 participants echoed this structural gap, reporting uncertainty about how to navigate referral processes. ‘I collaborate more with other professionals; few occupational therapists take optometry into account’ (optometrist, G1). ‘Follow‐up was impossible because the optometrist stopped contacting me’ (occupational therapist, G1). ‘Some nonoptometrist managers prioritise business over patient care, discouraging referrals’ (optometrist, G1).

#### 3.5.4. Role Awareness and Training Needs (G1 and G2)

G2 responses reflected uncertainty regarding the role of the other discipline, referral criteria and professional boundaries. A hospital‐based occupational therapist (G2) stated, ‘Collaboration is necessary, but I do not understand the role of the optometrist as a member of the rehabilitation team’. Both groups identified a need for further training, shared protocols and organisational support to enhance IPC.

## 4. Discussion

The findings of this study shed light on the current status of IPC between optometrists and occupational therapists in Spain. Such collaboration is particularly relevant, given the well‐documented link between visual function and occupational performance. By outlining facilitators and barriers, our research directly addresses a gap in the Spanish IPC literature. Although current practices remain fragmented, collaborators report positive experiences and high satisfaction, suggesting an encouraging outlook. These findings align with previous research on IPC between occupational therapists and optometrists in paediatric vision care, which described positive experiences and perceived improvements in client outcomes [20].

### 4.1. Fields of Practice and Collaboration With Other Professionals

The results underscore how opportunities for IPC differ across clinical settings. Respondents working in paediatrics reported more frequent collaboration. This finding may be related to high rates of visual difficulties among children with disabilities and neurodevelopmental disorders [4–6, 33]. These visual issues often affect occupational performance, such as reading and fine‐motor tasks, and may limit engagement in daily activities [5, 33]. These overlapping challenges may create natural opportunities for collaboration between optometrists and occupational therapists, enabling more tailored support, as participants noted.

Collaboration appears more limited in other areas of practice, such as low vision and ABI. In our sample, 131 participants (45.8%) worked with individuals with ABI; yet 77 (26.9%) were in G2, including 60 occupational therapists and 17 optometrists. This limited collaboration in ABI care may reflect the well‐documented underdiagnosis of visual impairments in this population [8, 9]. In addition, insufficient integration of vision specialists on rehabilitation teams, the lack of formal protocols and limited specialised training in vision represent structural barriers to the management of visual issues after ABI [7, 10, 11]. The 2025 European Stroke Organisation guidelines recommend the establishment of ‘close collaboration between stroke teams (particularly occupational therapy) and eye care teams’ [8]. Jointly developed interventions have shown potential to improve outcomes for individuals with ABI [19, 24]. Our findings reinforce the need for specific training and effective IPC to provide comprehensive care for this population.

Many G2 professionals in our sample reported working with individuals with low vision. Previous research found that over 58% of optometrists and ophthalmologists in the United States had never consulted or referred individuals with low vision to occupational therapists, and 82.4% had never received referrals from them [26]. Nonetheless, two recent studies illustrate the benefits of community‐based coordinated care between optometrists and well‐trained occupational therapists. The programme implemented in Florida showed improvements in functional performance and independence [2]. Likewise, the Seniors′ Eye Rehabilitation (SEER) programme reported reduced participation restrictions and improved well‐being [3]. A recent study projects a significant increase in the number of individuals with low vision in Spain due to ageing and chronic diseases, underscoring the need for integrated care models [1]. Further research is required to identify factors that may explain the limited collaboration in low‐vision care in Spain.

Optometrists providing vision therapy were more highly represented in G1 (*p* = 0.033). Vision therapy is gaining interest among Spanish optometrists; it still faces barriers, particularly a lack of recognition and prestige in eye care [16]. Despite evidence supporting its effectiveness [34, 35], limited recognition may hinder integration into broader care models. Interprofessional initiatives such as the ‘OT‐Led Remedial Vision Program’ have shown promising results by improving visual function and occupational performance [19]. Nevertheless, this model requires both expert optometric guidance and well‐trained occupational therapists [7].

### 4.2. Analysis of D′Amour Dimensions

G2 participants placed considerable importance on most IPC indicators, although not all components were valued equally. In contrast, G1 participants reported a moderate level of IPC enactment in practice, suggesting that many operate at the network or coordination level rather than in genuine collaboration or teamwork [21, 28], which is consistent with the lower scores in several indicators.

The *Shared Goals and Vision* dimension also showed room for improvement. Respondents from G1 indicated that goals are often set individually, despite evidence supporting joint goal‐setting and client involvement in decision‐making [12–14]. Differences in client‐centred practices, observed in both groups, are consistent with previous reports of greater integration within occupational therapy [17]. Moreover, our findings are consistent with recent research [22], which noted the need to develop client‐centred competencies among Spanish optometrists. Strengthening these skills could help build more comprehensive care and may improve client outcomes.

On the other hand, the *Internalisation* dimension received the highest scores across both groups, highlighting the importance of mutual acquaintanceship and trust in building collaborative teams and interprofessional networks [13, 21, 27]. Nevertheless, our results suggest that participants without prior experience identified professional silos as a potential barrier to establishing collaboration. Moreover, occupational therapists in our sample perceived optometrists as more protective of their professional boundaries. As a recent scoping review points out, role ambiguity and blurred boundaries may lead to defensive positioning [36], particularly when a discipline perceives limited recognition or faces structural constraints [37]. In Spain, optometrists are currently seeking greater clinical recognition [16], whereas occupational therapists continue to navigate identity consolidation and role confusion [15]. Further research is needed to explore these dynamics.

In G1, high ratings for ‘information exchange’ (median = 4) and low ratings for ‘formalisation tools’ (median = 2.5) suggest that collaborators share clinical information effectively. This feedback supports client follow‐up and is recognised as a key component in building trusting relationships, even in the absence of formal agreements; however, collaboration remains fragile without further formalisation [27, 36]. In addition, uncertainty persists regarding referral pathways in both groups, a factor identified in several countries as a systemic barrier [10, 11, 25, 26]. Even within networking dynamics, predefined referral pathways are recognised as essential for ensuring care continuity [28].

The *Governance* dimension received low ratings in G1, suggesting that IPC relies more on individual initiative and emergent leadership than on organisational support, as described by D′Amour et al. [27]. Professionals in G2 placed less importance on some organisational factors, such as ‘centrality’ (strategic direction) and ‘support for innovation’ (resources and training), as drivers of IPC. As Dib and Belrhiti emphasise, the lack of such support reduces collaboration to a mere individual effort, reinforcing professional silos and care fragmentation [36]. In this context, as collaboration seems more common in the private sector, formal agreements may promote cross‐sector cooperation [10, 12, 27]. Consequently, organisations should foster ‘connectivity’ by formally allocating scheduled time and spaces for collaboration, as these constraints remain a key barrier [13, 36, 37]. Health policies should prioritise governance models and shared pathways to ensure sustainable, integrated care across settings.

### 4.3. From Theory to Practice: Developing the Knowledge and Skills for Successful Collaboration

Although participants reported relatively high perceived knowledge of each other′s professions, the data revealed instances of failed collaboration and inconsistencies between perceptions and practice. This reflects the idea that IPC does not arise spontaneously but instead requires ongoing efforts at both organisational and individual levels, supported by targeted training and capacity‐building [13, 14, 36]. Our participants identified the need for advanced, practice‐oriented training to manage complex client needs and support collaboration between professionals, echoing findings in previous research [7, 20, 25].

Therefore, interprofessional education (IPE) is crucial to equip professionals with the skills needed for real‐world collaboration. Such investment is important, as working alongside other professionals may be insufficient to ensure meaningful and sustained partnerships. For example, Bell et al. [38] demonstrated that a multimodal IPE model enhanced occupational therapy students′ competencies in low‐vision care through collaboration with optometrists and ophthalmologists. Furthermore, practical IPE methods, such as role‐playing and case‐based discussions, promote mutual understanding, consolidate professional identity and clarify role ambiguity [39, 40].

Based on these insights, we recommend integrating structured IPE programmes in health professional education and continuing professional development in Spain. Such programmes could foster collaboration between optometrists and occupational therapists, improving client‐centred and integrated care services.

## 5. Limitations and Future Directions

This study has several limitations. Convenience and snowball sampling reduce the generalisability of the findings and may have introduced selection bias. Voluntary participation may have resulted in response bias, and self‐reported responses may be influenced by social desirability bias. Although a post hoc analysis estimated a margin of error of ±5.77% (95% confidence level) relative to the 2023 total population of registered occupational therapists and optometrists in Spain [41], these figures do not represent the entire practising workforce. Therefore, representativeness cannot be assumed. The sections assessing IPC indicators were developed for this study without separate psychometric validation; therefore, the results should be interpreted with caution. The data are specific to the Spanish context and may not be transferable to other countries.

Including G2 participants helped identify potential areas for collaboration; however, some in this group may not serve clients who need input from both professions, artificially inflating G2′s size. The data were not tied to particular organisations and may miss setting‐specific differences. Networking, coordination, collaboration and teamwork differ in task integration, role interdependence and shared responsibility [21, 28]. The study design makes it difficult to capture these nuances and the complex IPC dynamics.

Future studies should use focus groups and other qualitative methods to further explore IPC among occupational therapists and optometrists. Including the voices of clients and families is essential to better understand the real‐world impact of IPC on care. Research should also evaluate whether IPE and targeted training in Spain improve IPC practice and service delivery for people with visual impairment.

## 6. Conclusion

This study provides valuable insights into the experiences and challenges of IPC between optometrists and occupational therapists in Spain. Promising examples in paediatric care and vision therapy highlight its potential, yet collaboration remains limited in key areas such as low vision and ABI rehabilitation. Differences across settings and professions reflect persistent barriers, including role misconceptions and uncertainty about when and how to refer clients. Findings based on D′Amour′s indicators emphasise the importance of trust and mutual awareness as foundations for effective IPC. Still, gaps in formalisation, shared goal‐setting and organisational support often constrain IPC to basic networking or coordination, falling short of integrated teamwork. Addressing these challenges would benefit from structured IPE, clearer referral pathways and stronger professional networks. Enhancing collaboration between these professions presents a clear opportunity to improve client‐centred care, reduce fragmentation and optimise services for individuals with visual impairments and related functional difficulties. Achieving this will depend on coordinated efforts from organisations, practitioners, educators, professional bodies, policymakers and individuals with lived experience of visual impairment to foster a culture of interprofessional care across practice settings in Spain.

## Author Contributions

Supervision and conceptualisation: M.J.L‐F. and C.L‐F. Methodology: M.J.L‐F., C.L‐F., V.B., L.G‐C. and C.B. Formal analysis: M.J.L‐F., C.L‐F. and V.B. Investigation: M.J.L‐F. and C.L‐F. Data curation: M.J.L‐F., C.L‐F., V.B. and C.B. Writing—original draft preparation: M.J.L‐F., C.L‐F. and V.B. Writing—review and editing: M.J.L‐F., C.L‐F., V.B., L.G‐C. and C.B. Visualisation: M.J.L‐F., C.L‐F. and V.B.

## Funding

No funding was received for this manuscript.

## Disclosure

All authors have read and agreed to the published version of the manuscript.

## Conflicts of Interest

The authors declare no conflicts of interest.

## Supporting information


**Supporting Information** Additional supporting information can be found online in the Supporting Information section. Supporting Information. The file titled ‘SuppMat_Survey_IPC_Optometrists_Occupational Therapists_Spain’ contains the complete study questionnaire in its original Spanish version alongside an English translation.

## Data Availability

All data supporting the findings of this study are available from the corresponding author upon reasonable request.
