# Supplementary material for: Experiences and Perceptions of Interprofessional Collaboration Among Spanish Optometrists and Occupational Therapists: A Cross‐Sectional Study
Source: Occup Ther Int. 2026 Aug 10;2026:7088426. doi: 10.1155/oti/7088426 (PMC13458022; doi:10.1155/oti/7088426)
Supplement: Supplementary file 1 [file OTI-2026-7088426-s001.docx]

**Supplementary material**

**Encuesta: Colaboración Interprofesional entre Optometristas y Terapeutas Ocupacionales en España.**

*Survey: Interprofessional Collaboration between optometrists and occupational therapists in Spain*

*[Original in Spanish with English Translation]*

Desde la Universidad de Zaragoza estamos realizando un estudio sobre la colaboración entre ópticos-optometristas y terapeutas ocupacionales que trabajan en España. Le agradeceríamos que respondiera a este **cuestionario anónimo**, cuya participación es voluntaria y puede contribuir a una mejor comprensión de cómo esta colaboración impacta en la salud y el bienestar de la población a la que atienden. Para cumplir con las normas éticas de investigación, es necesario que otorgue su **consentimiento informado** antes de comenzar. Si tiene alguna duda o comentario, puede ponerse en contacto con el equipo investigador escribiendo a: [mjolopez@unizar.es](mailto:mjolopez@unizar.es) o [carmenlf@unizar.es](mailto:carmenlf@unizar.es). Para evitar respuestas duplicadas y permitirle retomar el cuestionario en caso de interrupción, se requiere **iniciar sesión en Google**. Esto no compromete su anonimato, ya que no se recopilarán datos personales asociados a su cuenta. Puede consultar la política de privacidad de Google en el siguiente enlace: <https://policies.google.com/privacy>. **Las preguntas marcadas con asterisco (*) son obligatorias.** El tiempo estimado para completar este cuestionario es de **15 minutos**. Por favor, recuerde hacer clic en **ENVIAR** al finalizar para registrar sus respuestas. ¡Muchas gracias por su colaboración!

[We are conducting a study at the University of Zaragoza on the collaboration between **optometrists and occupational therapists working in Spain**. We kindly invite you to complete this **anonymous questionnaire**, as your participation can contribute to a better understanding of how collaboration between these professionals impacts the health and well-being of the people they serve. In compliance with ethical research standards, you are required to provide your **informed consent** before proceeding. If you have any questions or comments, please contact the research team at: [mjolopez@unizar.es](mailto:mjolopez@unizar.es) or [carmenlf@unizar.es](mailto:carmenlf@unizar.es). To avoid duplicate submissions and allow you to resume the questionnaire if needed, **Google login is required**. Please note that your anonymity is guaranteed, as no personal data linked to your account will be collected. You can review Google’s privacy policy here: <https://policies.google.com/privacy>. **Questions marked with an asterisk (*) are mandatory.** Estimated completion time: **15 minutes**. Please remember to click **SUBMIT** at the end to record your responses. Thank you very much for your participation!]

¿Da su consentimiento expreso para participar en esta investigación? *(Do you give your explicit consent to participate in this research?)*

- Sí (ir a la sección 1)- Yes *(go to section 1)*
- No (ir a enviar el formulario)- No *(go to the end of the form)*

1. **Sección 1. Datos Sociodemográficos** *(Section 1. Sociodemographic Data)*
   1.1. **Profesión.** Seleccione su profesión principal. *(Please select your primary profession.)*

- Óptico-optometrista *(Optometrist)*
- Terapeuta ocupacional *(Occupational Therapist)*
  1.2. **Género.** Seleccione una opción. *(Please select one option.)*
- Mujer *(Female)*
- Hombre *(Male)*
- Prefiero no decirlo *(Prefer not to disclose)*
  1.3. **Edad.** Indique su edad en años. *(Please indicate your age in years.)*
       [Respuesta abierta /Open response]
  1.4. **Nivel académico más alto alcanzado.** Seleccione una opción. *(Please select your highest academic qualification.)*
- Diplomatura *(Diploma of Higher Education)*
- Grado *(Bachelor's Degree)*
- Máster *(Master's Degree)*
- Doctorado *(Doctoral Degree)*
- Otro, especifique: _______ *(Other, please specify: _______)*
  1.5. **Años de experiencia profesional.** Seleccione una opción. *(Please select your years of professional experience.)*
- Menos de 5 años *(Less than 5 years)*
- Entre 5 y 10 años *(5 to 10 years)*
- Entre 10 y 15 años *(10 to 15 years)*
- Entre 15 y 20 años *(15 to 20 years)*
- Más de 20 años *(More than 20 years)*
  1.6. **Ámbitos de trabajo actuales.** Seleccione todas las opciones que correspondan.
       *(Please select all that apply to your current work setting.)*
- Hospital *(Hospital)*
- Clínica privada *(Private Clinic)*
- Centro de atención visual *(Visual care centre or Optical store)*
- Centro de atención a personas mayores *(Aged care facility)*
- Organización sin ánimo de lucro *(Non-profit Organisation)*
- **Centro educativo o servicio de orientación (escuela, colegio, instituto).** (School or educational guidance service – e.g., School, high school, or counselling service)
- Centro de atención temprana *(Early intervention centre)*
- Universidad *(University)*
- Otro, especifique: _________ *(Other, please specify: _______)*

2. **Sección 2. Ámbitos de práctica** *(Section 2. Fields of Practice)*

**2.1. Ámbitos de atención o especialidad.** Seleccione todas las áreas en las que trabaja actualmente. *(Please select all the areas in which you currently practice.)*

**Optometristas *(Optometrists)***

- Baja visión *(Low vision)*
- Trastornos del aprendizaje *(Learning disabilities)*
- Daño cerebral adquirido *(Acquired brain injury)*
- Niños/as con discapacidad *(Children with disabilities)*
- Terapia visual *(Vision therapy)*
- Disfunción visual binocular *(Binocular vision dysfunction)*
- Estrabismo *(Strabismus)*
- Adaptación de lentes de contacto *(Contact lens fitting)*
- Otro, especifique: _______ *(Other, please specify: _______)*

**Terapeutas ocupacionales *(Occupational Therapists)***

- Baja visión *(Low vision)*
- Trastornos del aprendizaje *(Learning disabilities)*
- Daño cerebral adquirido *(Acquired brain injury)*
- Niños/as con discapacidad *(Children with disabilities)*
- Trastornos del neurodesarrollo *(neurodevelopmental disorders)*
- Enfermedades neurodegenerativas *(Neurodegenerative diseases)*
- Trastornos de salud mental *(Mental health disorders)*
- Otro, especifique: _______ *(Other, please specify: _______)*

**2.2. Grupos de edad de los clientes o pacientes con los que trabaja habitualmente.** Seleccione todas las opciones que correspondan. *(Please select all the age groups you commonly work with.)*

- Población pediátrica *(Paediatric population – children)*
- Adolescentes (>16 años)/Adultos *(Adolescents* *(>16 years)/Adults)*
- Personas mayores *(Older adults)*

**2.3. ¿Con qué otros profesionales colabora habitualmente?** *(Please select all the* ***other professionals*** *you usually collaborate with.)*

**Nota:** No incluya en esta pregunta a ópticos-optometristas ni a terapeutas ocupacionales. *(Note: Do not include optometrists or occupational therapists in this question.)*

- Oftalmólogos/as *(Ophthalmologists)*
- Psicólogos/as *(Psychologists)*
- Fisioterapeutas *(Physiotherapists)*
- Logopedas *(Speech therapists)*
- Trabajadores/as sociales *(Social workers)*
- Médicos/as de distintas especialidades médicas *(Physicians)*
- Personal de enfermería *(Nurses)*
- Maestros/as o Educadores/as *(Teachers or educators)*
- Otro, especifique: _______ *(Other, please specify: _______)*

**2.4. Colaboración actual o pasada con profesionales de la otra disciplina**

¿Ha colaborado alguna vez con un/a óptico-optometrista o terapeuta ocupacional?
*(Have you ever collaborated with an optometrist or an occupational therapist?)*

- Sí (ir a la sección 3). *(Yes-go to section 3)*
- No (ir a la sección 5). *(No-go to section 5)*

3. **Sección 3. Detalles de la colaboración (solo para participantes que han colaborado – G1).** *(Section 3. Collaboration Details – for participants with collaboration experience – G1 only)*

**3.1. Número de terapeutas ocupacionales u optometristas con los que ha colaborado.** *(How many occupational therapists or optometrists have you collaborated with?)*

- 1
- 2
- 3
- 4
- 5
- Más de 5 *(More than 5)*

**3.2. ¿Cómo comenzó la colaboración? (puede seleccionar varias opciones).** *(How did the collaboration start? Please select all that apply.)*

- Atendimos a un paciente en común *(By treating a patient together)*
- Trabajábamos en el mismo centro *(We worked in the same centre)*
- Otro, especifique: Formación, congresos o asociaciones de pacientes *(Other, please specify: training, conferences, or patient associations)*
  3.3. **¿Cuántos años lleva colaborando con profesionales de la otra disciplina?** *(How many years have you been collaborating with professionals from the other discipline?)*
- Menos de 2 años *(Less than 2 years)*
- Entre 2 y 5 años *(2 to 5 years)*
- Entre 5 y 10 años *(5 to 10 years)*
- Más de 10 años *(More than 10 years)*
  3.4. **Frecuencia de uso de los siguientes medios de comunicación durante la colaboración.** (How frequently do you use the following communication channels? Please rate each one from 1 to 5.)

**Escala / Scale:** 1 = Nunca (*Never*); 2 = Casi nunca (*Rarely*); 3 = A veces (*Sometimes*); 4 = Frecuentemente (*Frequently*); 5 = Siempre (*Always*)

Teléfono *(Phone calls)*

Mensajería instantánea (p. ej., WhatsApp) *(Instant messaging, e.g., WhatsApp)*

Correo electrónico *(Email)*

Reuniones informales (en el pasillo) *(Informal meetings, e.g., hallway chats)*

Reuniones programadas *(Scheduled meetings)*

A través de informes *(Through written reports)*

3.5. Grado de **satisfacción con la colaboración**. (How satisfied are you with your *collaboration experience?)*

1 = Muy insatisfecho/a *(Very dissatisfied)*
2 = Insatisfecho/a *(Dissatisfied)*
3 = Neutral *(Neutral)*
4 = Satisfecho/a *(Satisfied)*
5 = Muy satisfecho/a *(Very satisfied)*

3.6. ¿Cómo colabora habitualmente **con el otro profesional en su equipo? (puede seleccionar varias opciones).** *(How do you usually collaborate with the other professional in your team? Please select all that apply.)*
- Se derivan pacientes desde uno a otro centro *(Patients are referred from one centre to another)*
- Se derivan pacientes dentro del mismo centro *(Patients are referred within the same centre)*
  3.7. **Pensando en un caso** **reciente o memorable, ¿cómo cree que el uso de un enfoque de colaboración interprofesional optometrista/terapeuta ocupacional afectó en los resultados del paciente?** *(Thinking of a recent or memorable case, how do you think the use of an interprofessional optometrist/occupational therapist collaboration approach affected the patient’s outcomes?)*

[Respuesta abierta / Open response]

3.8. **¿Cómo le ha cambiado como profesional su colaboración con el optometrista/terapeuta ocupacional?** *(How has your collaboration with the optometrist/occupational therapist changed you as a professional?)*

[Respuesta abierta / Open response]

4. **Sección 4. Colaboración Interprofesional (basado en el Modelo de D’Amour - G1).** *(Section 4. G1-Interprofessional Collaboration Indicators – Based on D’Amour’s Model)*

A continuación, encontrará una serie de afirmaciones relacionadas con su **experiencia de colaboración interprofesional** entre terapeutas ocupacionales y optometristas. Se le pide que **indique en qué medida estos aspectos están presentes en su práctica diaria**. *(Instructions: Below you will find a series of statements related to your* ***experience of interprofessional collaboration*** *between occupational therapists and optometrists. Please indicate* ***to what extent these aspects are present in your daily practice)***

**Dimensión: Metas compartidas y visión común (Shared Goals and Vision)**

Por favor, indique con qué **frecuencia** estos aspectos están presentes en su práctica diaria cuando colabora con el otro profesional. *(Please indicate how often these aspects are present in your daily practice when collaborating with the other professional.)*

*1=Nunca (Never); 2=Rara Vez (Rarely); 3=A veces (Sometimes), 4=Frecuentemente (Frequently), 5=Siempre/Casi Siempre (Always/Almost Always).*

**Indicador 1. Objetivos compartidos *(Indicator 1. Shared Goals)***

1. Establecemos objetivos comunes. *(We set shared goals.)*
2. Tomamos decisiones comunes. *(We make decisions together.)*

**Indicador 2. Centrado en el cliente *(Indicator 2. Client-*** ***centred Orientation)***

3. Tenemos en cuenta las necesidades y preferencias de los pacientes/familias cuando colaboramos con el otro profesional. *(We take into account the needs and preferences of patients and families when collaborating with the other professional.)*
4. En su caso, ¿quién establece los objetivos de la intervención? marque todas las que correspondan. *(In your practice, who establishes the intervention goals? Select all that apply.)*

- Como profesional, establezco los objetivos de la intervención *(As a professional, I define the intervention goals.)*
- Los objetivos se establecen entre varios profesionales, trabajen o no en mi centro *(The goals are defined collaboratively among several professionals, whether or not they work in my centre.)*
- La familia o el cliente define los objetivos de la intervención *(The family or the client defines the intervention goals.)*

**Dimensión: Internalización (Internalisation)**

**Escala / Scale:**
1 = Nada en absoluto (Not at all); 2 = Poco (A little); 3 = Moderadamente (Moderately)
4 = Bastante (Quite a bit); 5 = Completamente (Completely)

**Indicador 3. Conocimiento Mutuo (*Indicator 3. Mutual Acquaintanceship*)**

Por favor señale hasta qué punto… *(Please indicate to what extent…)*

5. Conoce el rol y las competencias del otro profesional. (***To what extent do you know the other professional’s role and competencies?)***

**Indicador 4. Confianza (*Indicator 4. Trust)***

6. ¿Hasta qué punto confía en el criterio del otro profesional? (To what extent do you trust the other professional’s professional judgement?)
7. [Protección del rol y de los límites profesionales] ¿Hasta qué punto considera que la protección del rol y de los límites profesionales por parte del otro profesional dificulta la colaboración? ([Role Protectionism & Professional Boundaries] To what extent does the other professional's protection of their role and professional boundaries hinder collaboration?)

**Dimensión: Formalización (Formalisation)**

**Por favor, señale hasta qué punto estos elementos están presentes en su práctica diaria cuando colabora con el otro profesional.** (Please indicate to what extent these elements are present in your daily practice when collaborating with the other professional.)

1 = No está presente (Not present at all); 2 = Poco presente (Slightly present)
3 = Moderadamente presente (Moderately present); 4 = Bastante presente (Quite present); 5 = Muy presente (Highly present / Fully integrated in practice)

**Indicador 5. Herramientas Formales (*Indicator 5*. *Formalisation Tools*)**

8. Disponemos de protocolos de derivación que facilitan el trabajo conjunto. (We have referral protocols that facilitate joint work.)
9. **Disponemos de acuerdos formales que definen cómo debe organizarse la colaboración entre terapeutas ocupacionales y optometristas.** (We have formal agreements that define how collaboration between occupational therapists and optometrists should be organised.)

**Indicador 6.** **Intercambio de Información *(Indicator 6. Information Exchange)***

10. Disponemos de sistemas de intercambio de información que permiten compartir información sobre los pacientes de manera formal y documentada (por ejemplo, informes clínicos, plataformas de registro). (We have information exchange systems that allow us to share patient information in a formal and documented way (e.g., clinical reports, shared platforms).)
11. La información que intercambiamos sobre los pacientes es estructurada y útil para hacer seguimiento clínico. (The patient information we exchange is structured and useful for clinical follow-up.)

**Dimensión: Gobernanza (Governance)**

Por favor, **indique hasta qué punto estos aspectos están presentes en su lugar de trabajo o entorno profesional**. *(Please indicate to what extent these aspects are present in your workplace or professional setting.)*

1 = No está presente (Not present at all); 2 = Poco presente (Slightly present); 3 = Moderadamente presente (Moderately present); 4 = Bastante presente (Quite present); 5 = Muy presente (Highly present).

**Indicador 7. Líneas Estratégicas *(Indicator 7. Centrality)***

12. Mi centro de trabajo o institución fomenta acuerdos con otros centros o profesionales y la participación en redes de profesionales. (My organisation promotes agreements with other organisations or professionals and participation in professional networks.)

**Indicador 8. Liderazgo Compartido *(Indicator 8. Shared Leadership)***

13. Mi centro de trabajo u organización facilita la toma de decisiones clínicas basadas en el consenso de los profesionales implicados *(My organisation facilitates clinical decision-making based on consensus among the professionals involved.)*
14. **El liderazgo se comparte entre ambos profesionales, de modo que ninguno ejerce más poder o control que el otro.** *(Leadership is shared between both professionals, with neither exerting more power or control than the other.)*

**Indicador 9. Apoyo a la innovación *(Indicator 9. Support for Innovation)***

15. Mi centro de trabajo u organización apoya la innovación mediante recursos o formación específica para la colaboración interprofesional. (My organisation supports innovation through resources or specific training for interprofessional collaboration.)
16. Mi centro de trabajo facilita el reparto de responsabilidades entre ambos profesionales. (My organisation facilitates the sharing of responsibilities between both professionals.)

**Indicador 10. Espacios de encuentro *(Indicator 10. Connectivity)***

17. En mi centro se destinan tiempos específicos para reuniones o actividades colaborativas. (Dedicated time is allocated in my workplace for meetings or collaborative activities.)
18. **Contamos con espacios de encuentro y canales de comunicación para realizar reuniones (presenciales o virtuales) que facilitan el contacto entre profesionales.** (We have dedicated spaces for discussion and communication channels that facilitate contact between professionals and enable in-person or online meetings.)
5. **Sección 5. Colaboración interprofesional (basada en el modelo de D’Amour) (Group 2)** *(Section 5. G2-Interprofessional Collaboration – Based on D’Amour’s Model)*

**5.1. Por favor, señale hasta qué punto conoce las competencias y funciones del otro profesional (optometrista-terapeuta ocupacional).** *(Please indicate to what extent you know the other professional’s role and competencies.)*

1 = Nada en absoluto *(Not at all)*
2 = Poco *(A little)*
3 = Moderadamente *(Moderately)*
4 = Bastante *(Quite a bit)*
5 = Completamente *(Completely)*

**5.2.** [Protección del rol y de los límites profesionales] Por favor, señale hasta qué punto considera que la protección del rol y de los límites profesionales por parte del otro profesional dificulta la colaboración**.** *([Role Protectionism & Professional Boundaries] Please indicate to what extent you consider* that the other professional's protection of their role and professional boundaries hinders collaboration.*)*

1 = Nada en absoluto *(Not at all)*
2 = Poco *(A little)*
3 = Moderadamente *(Moderately)*
4 = Bastante *(Quite a bit)*
5 = Completamente *(Completely)*

**5.3. Por favor,** señale hasta qué punto está de acuerdo en que las siguientes acciones pueden facilitar la colaboración interprofesional **entre terapeutas ocupacionales y optometristas.** *(Please indicate to what extent you agree that the following actions could facilitate interprofessional collaboration between occupational therapists and optometrists.)*

1 = Totalmente en desacuerdo *(Strongly disagree);* 2 = En desacuerdo *(Disagree);* 3 = Ni de acuerdo ni en desacuerdo *(Neutral);* 4 = De acuerdo *(Agree);* 5 = Totalmente de acuerdo *(Strongly agree).*

**Afirmación / Statement**

**Indicador 1. Objetivos Compartidos (*Shared Goals*)**

1. Establecer objetivos comunes. *(Set shared goals.)*
2. Tomar decisiones clínicas de forma conjunta. *(Make joint clinical decisions.)*

**Indicador 2. Centrado en el cliente (*Client-centred Orientation*)**

3. Tener en cuenta las necesidades y preferencias de los pacientes y familias en la colaboración. *(Consider patient and family needs and preferences in collaboration.)*
4. Los pacientes y familias deben liderar el establecimiento de los objetivos de la intervención. *(Patients and families should lead goal-setting.)*

**Indicador 3. Conocimiento Mutuo (*Mutual Acquaintanceship*)**

5. Conocer las competencias y funciones del otro profesional. *(Know the other professional’s role and competencies.)*

**Indicador 4. Confianza (*Trust*)**

6. La confianza mutua *(Mutual trust)*

**Indicador 5. Herramientas de Formalización (*Formalisation Tools*)**

7. Los protocolos de derivación facilitan la colaboración y el trabajo conjunto entre terapeutas ocupacionales y optometristas. (Referral protocols facilitate collaboration *and joint work* between occupational therapists and optometrists.)
8. Los acuerdos formales entre instituciones **son necesarios** para definir cómo organizar la colaboración entre profesionales. (Formal agreements between organisations are necessary to define how collaboration between professionals is organised.)

**Indicador 6. Intercambio de Información *(Information Exchange)***

9. Los sistemas de intercambio de información deben permitir compartir información sobre los pacientes de manera formal y documentada (por ejemplo, informes clínicos, plataformas de registro). (Information exchange systems should allow patient information to be shared in a formal and documented way (e.g., clinical reports, shared platforms).)
10. La información que se comparte entre terapeutas ocupacionales y optometristas debe ser útil para el seguimiento del paciente. (The information shared between occupational therapists and optometrists should be useful for patient follow-up.)

**Indicador 7. Líneas Estratégicas *(Centrality)***

11. El centro de trabajo o la institución debe fomentar acuerdos con otros centros o profesionales y la participación en redes de profesionales.
    (The workplace or organisation should promote agreements with other organisations or professionals and participation in professional networks.)

**Indicador 8. Liderazgo Compartido *(Shared Leadership)***

12. El centro de trabajo/institución debe facilitar la toma de decisiones clínicas consensuadas entre los profesionales implicados.
    (The workplace or organisation should facilitate consensus-based clinical decision-making among the professionals involved.)
13. El liderazgo debería compartirse entre los profesionales, sin que ninguno ejerza más poder que el otro.
    (Leadership should be shared among professionals, with no one exerting more power than another.)

**Indicador 9. Apoyo a la innovación (*Support for Innovation)***

14. El centro de trabajo/institución debe apoyar la innovación mediante recursos o formación específica para la colaboración interprofesional.
    (The workplace or organisation should support innovation through resources or specific training for interprofessional collaboration.)
15. El centro de trabajo/institución debe facilitar el reparto de responsabilidades entre ambos profesionales.
    (The workplace or organisation should facilitate the sharing of responsibilities between both professionals.)

**Indicador 10. Espacios de encuentro *(Connectivity)***

16. Debe haber tiempos dedicados a reuniones o actividades colaborativas.
    *(There should be dedicated time for meetings or collaborative activities.)*
17. Debe haber espacios de encuentro y canales de comunicación (presenciales o virtuales) que faciliten el contacto entre profesionales. *(There should be spaces for discussion and communication channels, whether in person or online, that facilitate contact between professionals.)*

**6. Sección 6. Comentarios y sugerencias** (Section 6. Comments and Suggestions)

**6.1 ¿Considera que sería útil desarrollar o mejorar protocolos de derivación entre terapeutas ocupacionales y optometristas?**
*(Do you think it would be useful to develop or improve referral protocols between occupational therapists and optometrists?)*

- Sí *(Yes)*
- No *(No)*

**6.2. Si lo desea, puede añadir cualquier comentario o sugerencia.** (Would you like to add any comments or suggestions?) [Respuesta abierta / Open response]
